# Hypokalemic Paralysis Revealing Primary Sjogren's Syndrome: A Case Report

**DOI:** 10.31729/jnma.8266

**Published:** 2023-09-30

**Authors:** Pitambar Khanal, Sandip Paudel, Subodh Chapagain, Saurav Thapa, Madan Gyawali

**Affiliations:** 1 Maharajgunj Medical Campus, Maharajgunj, Kathmandu, Nepal; 2 Manosamajik Apanga Bandi Hospital, Nakkhu, Lalitpur, Nepal; 3 National Academy of Health Sciences, Mahaboudha, Kathmandu, Nepal; 4 Department of Internal Medicine, Bakulahar Ratnanagar Hospital, Tandi, Chitwan, Nepal

**Keywords:** *case reports*, *hypokalemia*, *renal tubular acidosis*, *Sjogren's syndrome*

## Abstract

Sjogren's syndrome is a rare chronic autoimmune disease characterised by dry eyes and dry mouth due to autoimmune destruction of the lacrimal and salivary glands, which can occur concurrently with other autoimmune diseases such as rheumatoid arthritis, systemic lupus erythematosus, or thyroiditis. It can lead to renal complications such as interstitial nephritis and glomerulonephritis, with distal/ type 1 renal tubular acidosis which may result in life-threatening electrolyte imbalance. We present a case of a 35-year-old female who presented with complaints of multiple episodes of muscle weakness. Type 1 renal tubular acidosis was discovered to be the cause of her symptoms which lead to the subsequent diagnosis of Sjogren's syndrome. This is rare presentation of Sjogren's syndrome, and it poses a challenge to diagnosis. Early detection and diagnosis of Sjogren's syndrome might be difficult due to existing diagnostic criteria, which contributes to a higher likelihood of missed diagnosis.

## INTRODUCTION

Sjogren's syndrome is a chronic systemic autoimmune disease characterised by dry eyes and dry mouth due to autoimmune destruction of the lacrimal and salivary glands, which can occur with other autoimmune diseases such as rheumatoid arthritis, systemic lupus erythematosus, or thyroiditis.^1^ It can present a variety of extra-glandular manifestations. Among them, tubulointerstitial nephritis and renal tubular dysfunction leading to distal renal tubular acidosis are common renal manifestations.^2,3^ Rarely, distal renal tubular acidosis can cause muscle paralysis due to severe hypokalemia, necessitating potassium supplements and alkali therapy.^4^ In our reported case, the patient had multiple episodes of muscle weakness as the only presenting complaint.

## CASE REPORT

A 35-year-old female presented to the Emergency Department (ED) with progressive weakness in her limbs for a week. She had two similar episodes of weakness over the previous 2 years for which she had been admitted, evaluated, and managed as per need. She gave a history of previous weakness attributed to electrolyte derangement. She was also a known case of primary hypothyroidism under levothyroxine supplement. On examination, she was ill-looking, normotensive, and tachycardic with normal oxygen saturation. Power in all four limbs was 3/5 and sensory examination was intact. In the ED, hypokalemia was noted in baseline investigations, and the computed tomography (CT) head was normal. Her arterial blood gas (ABG) at the time of admission revealed hyperchloremic non-anion gap metabolic acidosis and a potassium level of 2.1 mEq/l. In the absence of gastrointestinal loss or diuretic use, we suspect renal tubular acidosis (RTA) as a probable cause of metabolic acidosis. Her electrocardiography (ECG) was normal and she had normal urea and creatinine. Ultrasonography abdomen was unremarkable, and there was no evidence of obstructive uropathy. She was treated with intravenous (IV) potassium which slowly corrected and magnesium level was also sent which came to be normal.

Subsequent ABG showed improvement and the patient was symptomatically better. Based on her past history and current reports, we also sent an extractable nuclear antigen antibodies (ENA) panel which revealed positive anti-Sjogren's-syndrome-related antigen A autoantibodies (anti-SSA), strongly positive Ro-52 recombinant and negative antinuclear antibody (ANA), negative anti-double-stranded deoxyribonucleic acid (anti-ds DNA), and anti-scleroderma antibody (Scl-70). An ophthalmology consultation was done and a Schirmer eye test revealed ocular dryness as only 3 mm of the strip was wet after 5 min. Thyroid functions were normal, and viral markers were also negative (hepatitis B, C, and human immunodeficiency virus (HIV)). After ruling out the other causes, the possibility of Sjogren's syndrome was considered.

With the constellation of symptoms and lab findings as described above, she was diagnosed with distal renal tubular acidosis secondary to primary Sjogren's syndrome (pSS) with hypokalemic paralysis. The patient was treated with potassium citrate 2 g daily, sodium bicarbonate 1 g twice daily, prednisolone 40 mg daily, and mycophenolate mofetil 500 mg twice daily and she became symptomatically better with treatment. On follow-up after 1 week, her potassium was found to be normal and muscle weakness was improved.

## DISCUSSION

Sjogren syndrome is a long-term autoimmune disease primarily affecting lacrimal and salivary glands with varied extra-glandular features. Renal involvement varies from 1-33% in the form of glomerulonephritis and interstitial nephritis which may lead to renal tubular acidosis.^2^ Distal renal tubular acidosis (dRTA) occurs in about 25% of patients with pSS but only 8% of patients with distal renal tubular acidosis also have a prior diagnosis of pSS.^5,6^ Usually, dRTA presents with mild hypokalemia and normal anion gap acidosis. However, very few cases reporting severe hypokalemia in dRTA leading to muscle paralysis have been published in the literature.

In our case, the patient had three episodes of muscle weakness for the last 2 years without other classical glandular symptoms of Sjogren syndrome (SS). Muscle weakness was initially attributed to levothyroxine-treated hypothyroidism, but when further lab results revealed hypokalemia, the patient's condition was further assessed. Then, hypokalemia was linked with normal anion gap renal tubular acidosis as evident in ABG findings. So, later on, an ENA profile was sent on which serological findings revealed positive findings for SS.

We established the diagnosis of pSS based on ACR-EULAR criteria for the diagnosis of Sjogren syndrome.^7^ Under-expression of H(+)-ATPase and pendrin is thought to be the main mechanism of distal renal tubular acidosis in patients with SS.^8^ Usually dRTA is mild in pSS but can be severe in some patients. Hypokalemia is a manifestation primarily due to dRTA but can occur without dRTA as well in patients with pSS. The mechanism of the latter is due to tubular injury induced by tubulointerstitial nephritis.^6^ Hypokalemia results in muscle weakness which is the presenting feature of our patient. The reason behind it is an alteration in the generation of an action potential. There are various causes of muscle weakness such as stroke, spinal cord diseases, peripheral nerve disease, neuromuscular junction disorder and myopathies. Because patients who present with muscle weakness are evaluated in the context of the above-mentioned causes, physicians rarely associate muscle weakness with SS at first glance, despite the fact that there have been a few reported cases of muscle weakness as a presenting feature of SS in the past.^9^

We focus on this case because it presented muscle weakness as the only presenting feature of pSS. So, clinicians focused on the above-mentioned causes and came to the conclusion with hypokalemia was the apparent cause after ruling out other causes. So, the patient was managed in line of correction of hypokalemia which resulted in the patient's temporary improvement of symptoms. But this also led to repeated episodes of muscle weakness in the patient because the underlying cause was not addressed due to the rarity of linkage between muscle weakness as the only presenting feature and pSS. As the patient had recurring episodes of weakness owing to hypokalemia, we looked for an underlying cause of hypokalemia, which led us to dRTA. Since SS is also a common cause of dRTA, we evaluated the patient with an ENA panel and Schirmer's test which led us to the diagnosis of SS although the patient did not have classical glandular manifestations.

We could not evaluate the patient for various channel defects that could cause hypokalemia. Although we linked hypokalemia in the patient to be due to dRTA, we could not rule out other channel defects that could be present along with dRTA.

Early detection and diagnosis of SS might be difficult due to existing diagnostic criteria, which contributes to a higher likelihood of missed diagnosis. Patients may come with a variety of symptoms that are incongruous with the disease's conventional presentation. Thus, patients who present with recurring/persistent hypokalemia should undergo additional diagnostic testing to rule out SS while also receiving prompt management of metabolic derangements with potassium and alkali treatment to avoid potentially fatal sequelae.
